# Rapid Solution-Based SERS Detection of Pesticides Using Graphene Oxide-Coated Silver–Gold Nanostars

**DOI:** 10.1021/acsanm.4c01122

**Published:** 2024-05-08

**Authors:** Supriya Atta, Tamer Sharaf, Tuan Vo-Dinh

**Affiliations:** a Fitzpatrick Institute for Photonics, Ain Shams University, Cairo, Egypt,; b Department of Biomedical Engineering, Ain Shams University, Cairo, Egypt,; c Department of Physics, Ain Shams University, Cairo, Egypt,; d Department of Chemistry, Duke University, Durham, NC 27708, USA.

**Keywords:** Gold nanostars, graphene oxide plasmonics, bimetallic, SERS, sensing, pesticides

## Abstract

This study presents the development of an affordable and straightforward solution-based surface-enhanced Raman scattering (SERS) detection platform, employing plasmonic-active silver-gold nanostars coated with graphene oxide (GO-SGNS). The inclusion of GO with SGNS produced several advantages, including colloidal stability, SERS detection reproducibility, and improved sensitivity. The solution-based SERS platform exhibited an ultralow detection capability with high reproducibility, and we achieved the limit of detection (LOD) as low as 10 pM, 50 pM, 100 pM, and 100 pM for the pesticides- ziram, phorate, triazophos, and azinphos-methyl, respectively. To illustrate the potential for food safety, the surfaces of apples were pretreated with pesticides and were directly analyzed without any sample pretreatment. Furthermore, the solution-based SERS platform provided rapid detection of the four pesticides in binary and quaternary mixtures on apple surfaces, indicating good feasibility and multiplex capability of the method.

## Graphical Abstract

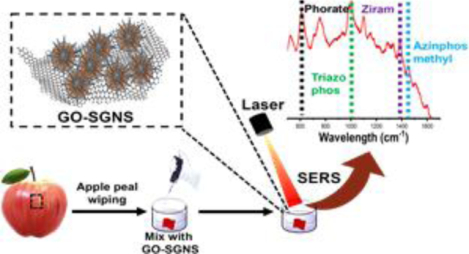

## INTRODUCTION

Pesticides are essential for increase the production of food in modern agricultural processes by protecting crops from insects, weeds, and fungi^1–3^. However, inappropriate, and excessive usage of pesticides causes food pollution, which could adversely affect human health and the environment. Therefore, it is essential to develop a rapid, sensitive, and cost-effective detection method to monitor pesticides and prevent their excessive usage. Several analytical methods have been reported to detect pesticides with high sensitivity and specificity, such as high-performance liquid chromatography (HPLC), gas chromatography (GC), and GC-MS^4–8^. However, these methods require time-consuming sample pretreatments, expensive experimental instrumentation, and special personnel to operate the instruments. Moreover, these methods are not readily adapted to detect multiple pesticides in real samples. Hence, there is increasing demand for the development of efficient analytical methods for rapid on-site detection of multiple pesticides.

Surface-enhanced Raman scattering (SERS) has emerged as a cost-effective rapid analytical tool because of the advantages of the portable feature and unique fingerprint information of a variety of target molecules, including pesticides^9–15^, drugs^16, 17^, biomarkers^18, 19^, and many other analytes^20–22^. More than three decades ago our laboratory first introduced the use of SERS as an analytical technique for trace organic analysis^23^ and has developed this technique for a wide variety of chemical and biosensing applications^24–26^. The SERS detection method is attributed to two main processes, the electromagnetic enhancement, which is associated with the surface plasmon resonance (SPR) in noble metal nanoparticles (such as gold or silver nanoparticles) and chemical enhancement, which is associated with charge transfer between the target molecules and SERS substrates^20^. Generally, it is believed that the local electric field enhancement is the main contributor to the SERS effect, which is dependent on the metal nanostructure. To improve the SERS sensitivity and stability, extensive studies have focused on optimizing plasmonic noble metal nanoparticles. Recently, anisotropic bimetallic gold-silver nanostructures have attracted significant attention in recent years to take the advantage of both the metal gold and silver, where silver facilitates higher enhancement factors in comparison to gold, and gold has better chemical stability and fine tunability of the morphology with a broader localized surface plasmon resonance (LSPR)^10, 27–30^. Various approaches have been devoted to developing gold-silver nanostructures with a variety of different shape morphologies, including spheres, cubes, rods, and stars, to achieve ultra-high SERS sensitivity^29, 31–33^. Among them, bimetallic gold-silver nanostars have attracted interest in the research community because of their unique morphology and optical properties^31–33^. There is great interest in improving the properties and performance of bimetallic gold-silver nanostars such as improved stability in real-world conditions.

For field application, a solution-based SERS detection platform has emerged as a facile and practical strategy for the real-time analysis of pesticide residues on real samples. However, the solution-based SERS analysis would fail to detect pesticides in real-life samples such as apple peels and leaves. Since the actual samples contain large amounts of macromolecules such as proteins, organic acids, and sugar, they adhere non-specifically to the gold nanoparticles and cause aggregation of nanoparticles, which could reduce the SERS sensitivity. To reduce complex matrix interference of the real samples, sample pretreatment liquid-liquid or solid-liquid separation method has been applied to purify the test material and eliminate these interferences^34^. However, these methods are unsuitable to become practical tools for point-of-need SERS detection of pesticides. Therefore, it is important to develop a nanomaterial system that is stable in real samples and detects analyte molecules without any sample preparation. To address this problem, surface coating of plasmonic nanoparticles with high surface area inert oxide material plays a significant role in maintaining the stability of the delicate plasmonic nanostructure, which can significantly improve the detection of analytes in real-world samples. Recently, GO had gained attention due to its unique properties, including high surface area, excellent chemical stability, and strong interaction with various molecules^35–39^. Additionally, the presence of GO can enhance the SERS signal through charge transfer, leading to overall amplification of the Raman signal^40–44^. These properties make GO a promising candidate for stabilizing bimetallic gold-silver nanostars morphology.

There are some reported methods for GO-coated gold nanoparticles for the detection of analytes^42, 45, 46^. However, in most cases, solid substrate preparation is necessary to detect analytes^42^, which is time-consuming and requires specialized skills. Also, solution-based SERS detection using GO-modified gold nanoparticles lacks rapid detection of analytes, which requires a long time to enrich the analytes on the surface of the nanoparticles^46^. Therefore, it is important to design a plasmonic nanomaterial with the maximum number of hot spots, such as GNS, and combine it with high SERS scattering efficiency material, such as silver. Further, hybrid plasmonic nanomaterials can be combined with GO to achieve ever brighter SERS enhancement.

**Figure 1.**
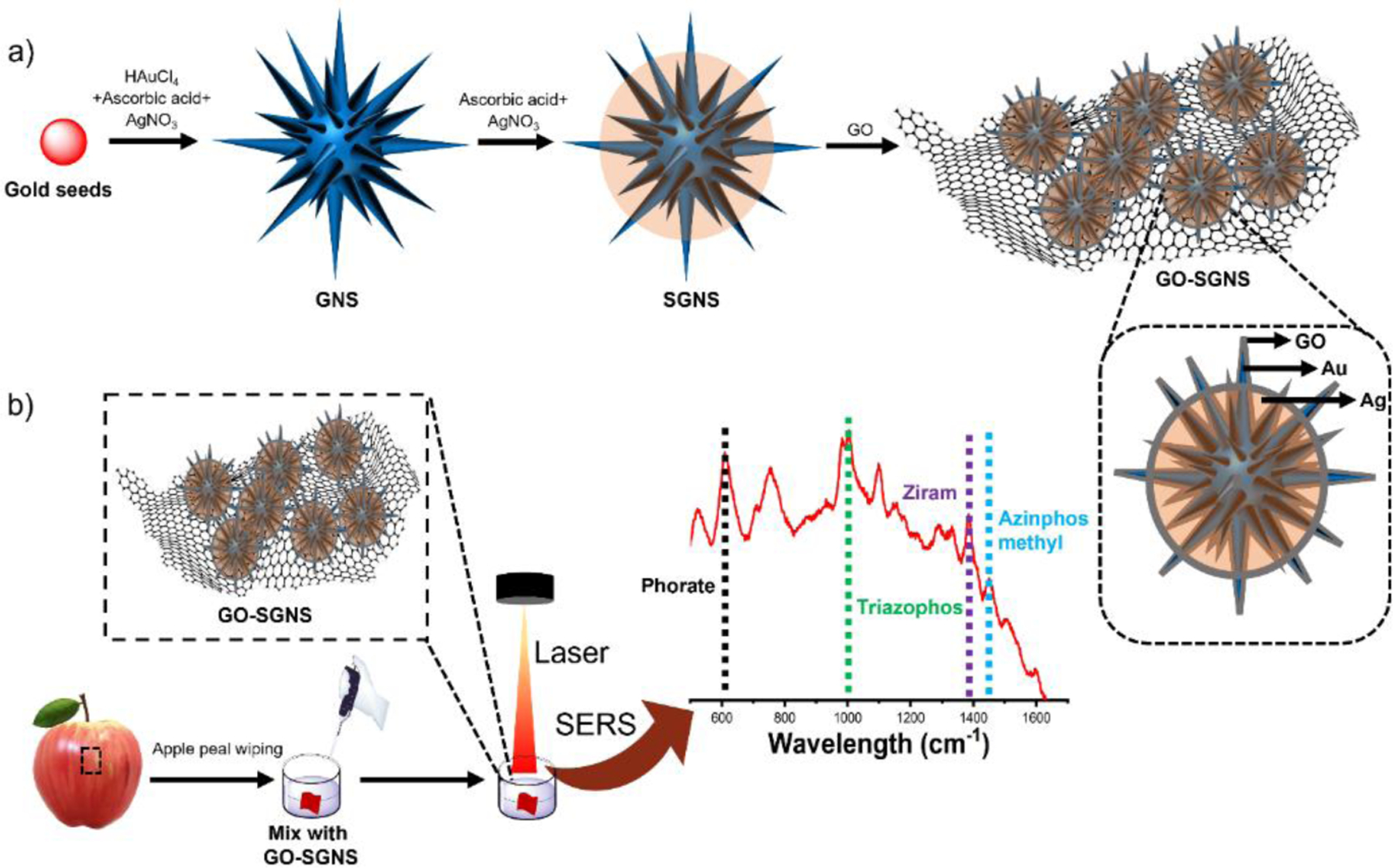
Schematic for GO-SGNS synthesis (a) and the solution-based SERS sensing platform for the multiplex detection of pesticides (b).

In this study, our strategy is to fabricate the SGNS nanostructure with GO to achieve stable and sensitive SERS signals. The outermost GO coating effectively protects the delicate sharp spikes of SGNS from aggregation, which results in improved reproducibility and stability of the SERS signal. The fabrication process of SGNS with GO and multiplex identification and quantitative analysis of pesticide residues is shown in Figure 1. The SERS sensor GO-SGNS exhibited high sensitivity, long-term stability, and high reproducibility. Moreover, we have applied our SERS sensor to directly detect pesticides on real sample surfaces such as apple surfaces. The results indicate that our SERS sensor GO-SGNS has produced an excellent SERS signal enhancement and illustrates its potential as a high-throughput SERS platform for rapid sensitive, quantitative detection of multiple pesticides.

## EXPERIMENTAL SECTION

### Materials and characterization

Chloroauric acid (HAuCl_4_), L-ascorbic acid, silver nitrate (AgNO_3_, 99.8%) hydrochloric acid (HCl), trisodium citrate (Na_3_C_6_H_5_O_7_), thiophenol, ziram, phorate, triazophos, and azinphos-methyl were purchased from Sigma-Aldrich. Milli-Q deionized (DI) water was used throughout the experiment. Graphene Oxide Water Dispersion (0.4 wt. % Concentration) was purchased from Graphenea. The TEM images of GNS, SGNS, and GO-SGNS were acquired using Thermo-Fisher Titan 80–300. UV-vis spectra were recorded using a Shimadzu UV-3600i spectrometer with cuvettes of 1 cm path length at room temperature.

### Synthesis of GNS, SGNS, and GO-SGNS

12 nm gold seeds were synthesized using our previously described method^47^. Brifefly, 7.5 mL of a 1% sodium citrate solution was added to 50 mL of a 1 mM AuCl_4_ aqueous solution under boiling conditions. Keeping the solution boiling for another 60 minutes, the color changes to a red color; after cooling to room temperature in an ice bath, it was stored at 2–4 °C until further use. GNS were synthesized by following previously reported method^48^. Briefly, 200 μL of 1 M HCl to a solution containing 50 mL of 1 mM HAuCl_4_ and 500 μL of Au seed solution. After that, we added a solution of 2 mL of 3 mM AgNO_3_ and 1 mL of 100 mM ascorbic acid to the solution and the solution was stirred for 2 minutes which was used further for the silver coating process.

SGNS was synthesized by following our previously reported method.^10^ Briefly, for SGNS’, 48 mL milli-Q water was added to the GNS solution first. Then, 1 mL 15 mM AgNO_3_ and 1 mL 100 mM ascorbic acid were added simultaneously to the GNS solution. To prepare SGNS”, 44 mL of milli-Q water was added to the GNS solution first. Then, 3 mL 15 mM AgNO_3_ and 3 mL 100 mM ascorbic acid were mixed simultaneously to the GNS solution. To prepare SGNS, 40 mL of milli-Q water was added to the GNS solution first. Then, five mL of 15 mM AgNO_3_ and five mL of 100 mM ascorbic acid were added simultaneously to the GNS solution. To grow the silver layer on the GNS, the solution was stirred overnight.

GO-SGNS was synthesized by the addition of GO solution directly on the SGNS solution. Briefly, 200 μL of GO solution (0.4 wt. % Concentration) was added to 100 mL of SGNS solution. The GO loading from 25 μL of GO to 1000 μL of GO solution (0.4 wt. % Concentration) was investigated. To minimize the Raman interference effect of GO, we combined GO (200 μL) with SGNS to make further SERS measurements. We have followed the same protocol for the synthesis of GO-SGNS’ and GO-SGNS”. The maximum GO loading capacity of SGNS was observed up to 0.0016% (wt. %), and SGNS was aggregated at above 0.0016% (wt. %) GO concentration. The solution was stirred overnight and stored at 2–8 °C.

### Raman measurements

Raman measurements were performed by using a laboratory build portable Raman instrument having a 785-nm laser source (Rigaku Xantus TM-1 handheld Raman device), a fiber optic probe (InPhotonics RamanProbe), a spectrometer (Princeton Instruments Acton LS 785), and a CCD camera (Princeton Instruments PIXIS: 100BR_eXcelon). The laser power of the Rigaku Xantus TM-1 was set at 200 mW, and exposure time was set at 1 sec.

### Practical application of the proposed SERS platform

For the detection of pesticides on the surface of an apple, the apples were cleaned with ultrapure water and absolute ethanol, and then, 100 μL of the as-prepared pesticide solution was spread onto an apple surface and dried at room temperature. Thereafter, the apple surface was cut into 1 × 1 cm squares and mixed with the GO-SGNS solution, and the following SERS study was performed according to the aforementioned steps.

## RESULTS AND DISCUSSION

Silver-coated gold nanoparticles can produce a much stronger SERS signal than gold nanoparticles because of the high scattering silver enhancing the overall electromagnetic field^27, 29, 31, 32, 49^. In addition, to achieve ultra-high SERS sensitivity, controlled assembly of gold-silver nanoparticles over GO nanosheets can further enhance the SERS signal because of the synergetic effect of both the electromagnetic enhancement induced by plasmonic SGNS nanoparticles and the chemical enhancement induced by GO^50^. Moreover, GO can produce a large contact area, create more “hot spots”, and protect the internal silver layer from oxidation. In this study, we have utilized sharply spiked gold nanostars coated with silver (SGNS), which can generate extremely strong electromagnetic fields between the adjacent plasmonic nanostructures via exciting the localized surface plasmon resonance (LSPR), and then the GO layer was decorated on SGNS nanostructure to further enhances the SERS signal via chemical enhancement (Figure 1a). Figure 1b shows the solution-based SERS detection platform based on GO-SGNS for multiple pesticide residue identification. Such a detection platform based on GO-SGNS is expected to exhibit highly improved SERS performance with a rapid and simultaneous screening of multiple pesticides.

### Morphological structure characterization of GO-SGNS

**Figure 2.**
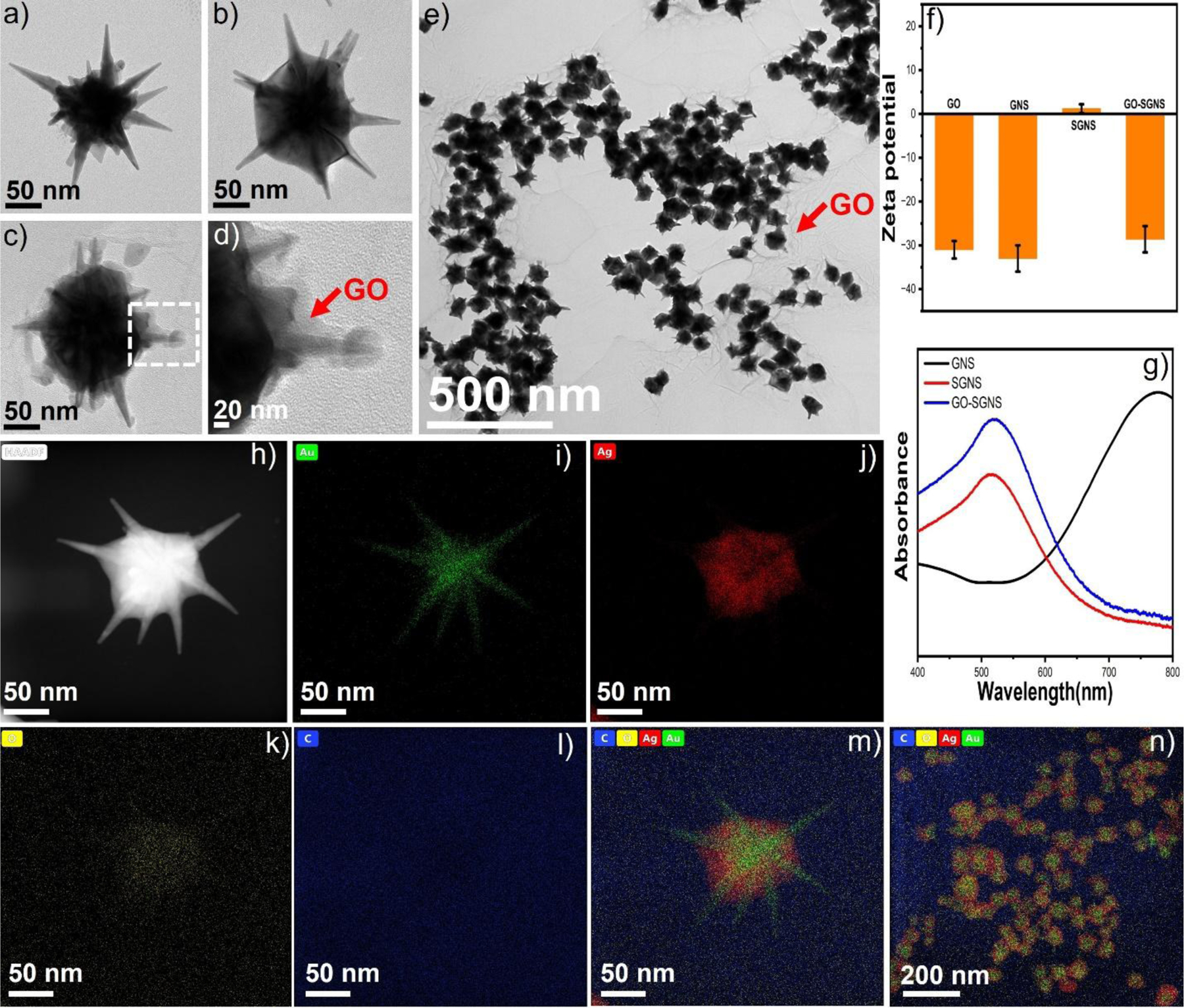
TEM images of GNS (a), SGNS (b), and GO-SGNS (c). Magnified TEM image of the GO-SGNS indicating that GO is covered on the spikes of SGNS (d). TEM image of GO-SGNS having multiple nanoparticles (e). The zeta-potential of GO, GNS, SGNS, and GO-SGNS (f). UV-vis spectra of GNS, SGNS, and GO-SGNS show that the maximum plasmon resonance of GNS was blue shifted from 770 nm to 515 nm and then red shifted from 515 nm to 522 nm after GO addition (g). The HAADF and EDS mapping of GO-SGNS with the elements of C, O, Au, and Ag (h-m). EDS mapping of multiple GO-SGNS nanoparticles with the elements of C, O, Au, and Ag (n).

In this study, we first synthesized GNS by following a seed-mediated surfactant-free synthesis method reported previously by our group.^51^ Figure 2a and Figure S1a displayed the TEM images of a typical GNS morphology. In the second step, GNS was coated with silver to achieve SGNS morphology. Figure 2b and Figure S1b showed the TEM image of SGNS, which exhibited an enlarged diameter of the core of about 120 nm, indicating the growth of a silver shell at the core of GNS. In the third step, SGNS was decorated with GO. We have confirmed the wrapping of SGNS with GO using TEM. The TEM image in Figure 2c–d showed a thin layer of GO on SGNS and multiple SGNS nanoparticles decorated on GO sheets. It is important to note that the GO is highly negatively charged due to the residual oxygen-containing functionalities^52^. The zeta potential of GO was measured to be around −31 mV, which indicates that, in order to bind with GO, gold nanoparticles should possess a positively charged surface. Interestingly, the zeta potential of GNS and SGNS was −33 mV and 1.2 mV (Figure 2f). An acidic medium was used to coat GNS with silver, i.e. adding AgNO_3_ to ascorbic acid at high concentrations, and the pH of the SGNS was measured to pH 2.1. It is important to note that the zeta potential value of the nanoparticles can increase with increasing the acidity of the medium and the concentration of AgNO_3_^53, 54^. Thus, we believe that the SGNS has a slightly higher zeta potential than GNS as the SGNS solution contains a high concentration of AgNO_3_ and ascorbic acid. As SGNS has a positively charged surface, SGNS was suitable for binding with negatively charged GO. The maximum GO loading capacity of SGNS was observed up to 0.0016% (wt. %), and SGNS was aggregated at above 0.0016% (wt. %) GO concentration. Moreover, the DLS size distribution shows that the GO-SGNS is highly uniform at 0.0016% (wt. %) GO concentration (Figure S1c). Furthermore, the UV-vis absorbance spectra indicated that the maximum plasmon resonance of GNS was blue shifted from 770 nm to 515 nm after silver coating. The maximum plasmon resonance of SGNS was red-shifted from 515 nm to 522 nm after GO addition indicating that the nanoparticle surface is decorated with GO (Figure 2g). The blue shifting of the plasmon resonance of GNS was observed for the silver coating on gold nanoparticles that was consistent with the reported values^10, 55^. Furthermore, energy dispersive X-ray spectroscopy (EDS) elemental mapping has been performed to analyze the elemental distribution of the GO-SGNS. Figure 2h–n displays the EDS mapping of GO-SGNSs with the elements of C, O, Au, and Ag. EDS images indicate that the Ag layer is preferentially deposited on the core of GNS, which agrees with previous reports^27, 55^. The silver thickness of SGNS’, SGNS”, and SGNS was determined from the TEM images (Figure S2), where the core-radius of GNS increased from SGNS’ to SGNS. The average silver thickness was 8 nm, 16 nm, and 30 nm, SGNS’, SGNS”, and SGNS respectively, which is consistent with the previously reported silver coated gold nanostars^56^.

### SERS enhancement of GO-SGNS

It is well-known that GO has signature Raman peaks at around 1350 cm^−1^ (D peak), and 158 cm^−1^ (G peak)^57^, which could interfere with the SERS signal of the target analytes. Our goal was to minimize the Raman interference effect of GO and maximize chemical enhancement of GO through the charge transfer effect by tuning different amounts of GO loaded onto the SGNS solution. Figure S3 shows that that the characteristic SERS signal of GO at 1330 cm^−1^ and 1584 cm^−1^ increased with increased GO loading from 25 μL of GO to 1000 μL of GO solution (0.4 wt. % Concentration). Interestingly, the SERS signal of GO at 1330 cm^−1^ appeared at above 200 μL of GO addition. We used the combination of GO (200 μL of GO) with SGNS for further SERS measurements of pesticides to minimize the Raman interference effect of GO.

**Figure 3.**
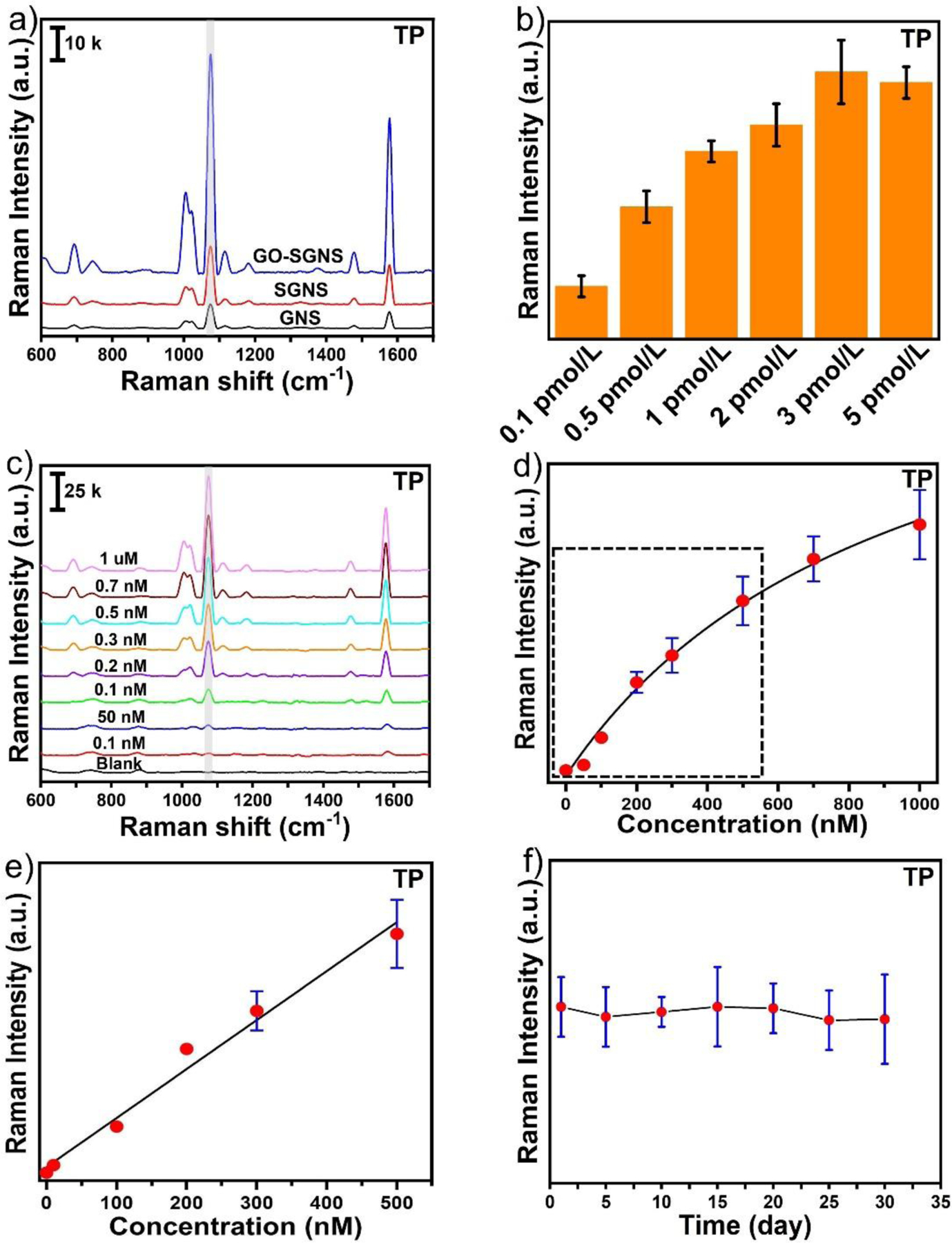
The SERS spectra of TP at the concentration of 100 nM with GNS (black), SGNS (red) and GO-SGNS (blue) which shows that GO-SGNS has the maximum SERS enhancement (a). The SERS intensity of TP at 1075 cm^−1^ with different concentrations of GO-SGNS (b). The SERS spectra and the corresponding calibration curve of TP at different concentrations ranging from 1μM to 0.1 nM with GO-SGNS (c-e). The reproducibility and stability of the GO-SGNS with time (f).

To validate the SERS performance of the hybrid nanostructure- GO-SGNS, we studied solution-based SERS measurements with thiophenol (TP) as a model analyte molecule using a portable Raman instrument. The typical SERS spectra of TP exhibited four characteristic peaks at 1003, 1025, 1075, and 1584 cm^−1^, which are assigned to the vibrational bands of in-plane C-C-C ring bending (ring breathing), in-plane C-H ring bending, in-plane C-C-C bending + C-S stretching, symmetric in plane C-C ring stretching, respectively (Figure 3a)^58^. Figure 3a displayed the SERS comparison of GNS, SGNS and GO-SGNS at 3 pmol/L concentration, indicating that the characteristic SERS peak of TP at 1075 cm^−1^ for SGNS was almost two times higher than GNS. More importantly, the characteristic SERS peak of TP at 1075 cm^−1^ for GO-SGNS was almost eight times higher than GNS and four times higher than SGNS. Interestingly, we observed that the characteristic SERS peak of TP at 1075 cm^−1^ for GO-SGNS was almost 2 times higher than GO-SGNS’, and 1.5 times higher GO-SGNS” (Figure S4). We believe that GO-SGNS exhibited the highest SERS enhancement which might be because of its high aspect-ratio sharp spikes as well as higher surface scattering silver layer, GO-SGNS shows the highest SERS enhancement than GO-SGNS’ and GO-SGNS”^10^. The high sensitivity is attributed to the synergistic effect of the charge transfer of GO and electromagnetic field enhancement from SGNS.

To obtain the best SERS enhancement conditions of the solution-based SERS detection platform, we optimized the concentration of GO-SGNS. Figure 3b exhibited the SERS peak intensity of TP at 1075 cm^−1^ at different concentrations of GO-SGNS ranging from 0.1 pmol/L to 5 pmol/L, where the concentration was calculated by following our previously reported procedure^59^. As shown in Figure 3b, the SERS intensity was increased with increasing the concentration of TP and reached a maximum value at 3 pmol/L before reaching a plateau. The above optimized experimental conditions were employed further for performing pesticide detection. Figure 3c displayed the SERS spectra of TP at different concentrations from 1 μM to 0.1 nM. The SERS peak of TP at 1075 cm^−1^ was chosen to establish the calibration curve of GO-SGNS. Figure 3d exhibited the calibration curve of TP, where the SERS intensity at 1075 cm^−1^ versus the concentration of TP was fitted with a one-site specific binding equation as described below:

$$y=\text{Bmax}\times \frac{x}{Kd+x}$$

Where *y* is the SERS intensity of TP, *Bmax* is the SERS intensity at the saturation coverage (Bmax= 143571), *Kd* is the equilibrium dissociation constant in the competitive adsorption process (*Kd* =943.5), and *x* is the concentration of TP. Moreover, Figure 3e displayed a linear relationship between the SERS intensity at 1075 cm^−1^ versus the concentration of TP in the concentration range from 0.1 nM to 0.3 nM. According to the calibration curve, the R^2^ value was 0.98. The LOD of TP was calculated to be 0.05 nM with a good signal-to-noise ratio (S/N= 3.5).

We further calculated the analytical enhancement factor (AEF) of the GO-SGNS by following the previously described method^66, 67^ as: AEF= (I_SERS_/C_SERS_)/ (I_Raman_/C_Raman_), where I_SERS_ and I_Raman_ are the SERS intensity of TP at 1584 cm^−1^ in the presence and in the absence of GO-SGNS, respectively. The C_SERS_ and C_Raman_ are the concentrations of TP in the SERS and normal Raman measurements, respectively. The Raman spectrum of TP at 100 mM concentration and the SERS spectra of TP at 0.1 nM concentration were displayed in Figure S5. The AEF of GO-SGNS was calculated as 3.2 × 10^8^. We compared the AEF value of GO-SGNS with the reported colloidal based SERS-sensing approaches (Table 1), which indicates that our SERS platform is more sensitive than the reported colloidal SERS substrates. The GO-SGNS contains spiky gold nanostars generating strong intense electric fields compared to other different sized or shaped gold nanoparticles. Hybrid plasmonic SGNS nanomaterials were also fabricated with GO to enhance the SERS signal through charge transfer, resulting in a higher SERS enhancement.

In addition to the outstanding quantitative SERS detection capability, uniformity and reproducibility are two critical indicators for the practical SERS substrate. For the uniformity measurement, the SERS signals of TP at 0.1 μM were collected from 30 spots of GO-SGNS. Figure S6 showed the SERS signals of TP collected from 30 spots. As depicted in Figure S6, the relative standard deviation (RSD) value of the SERS peak intensity of TP at 1075 cm^−1^ was calculated to be 5.5 %, underscoring the high reproducibility of the solution-based SERS platform. Moreover, the stabilities of GO-SGNS solution with different storage times at 2–8 °C were also investigated. Figure 3f displayed that the SERS signal of TP at 1075 cm^−1^ at 0.1 μM was almost the same after 30 days of storage, indicating that the SGNS anchored on GO exhibited long stability and protected the silver layer from oxidation. These results illustrate that the GO-SGNS has excellent detection sensitivity and quantitative capability, providing favorable conditions for rapid point-of-need on-site application of solution-based SERS substrate.

### SERS detection of pesticides in water

**Figure 4.**
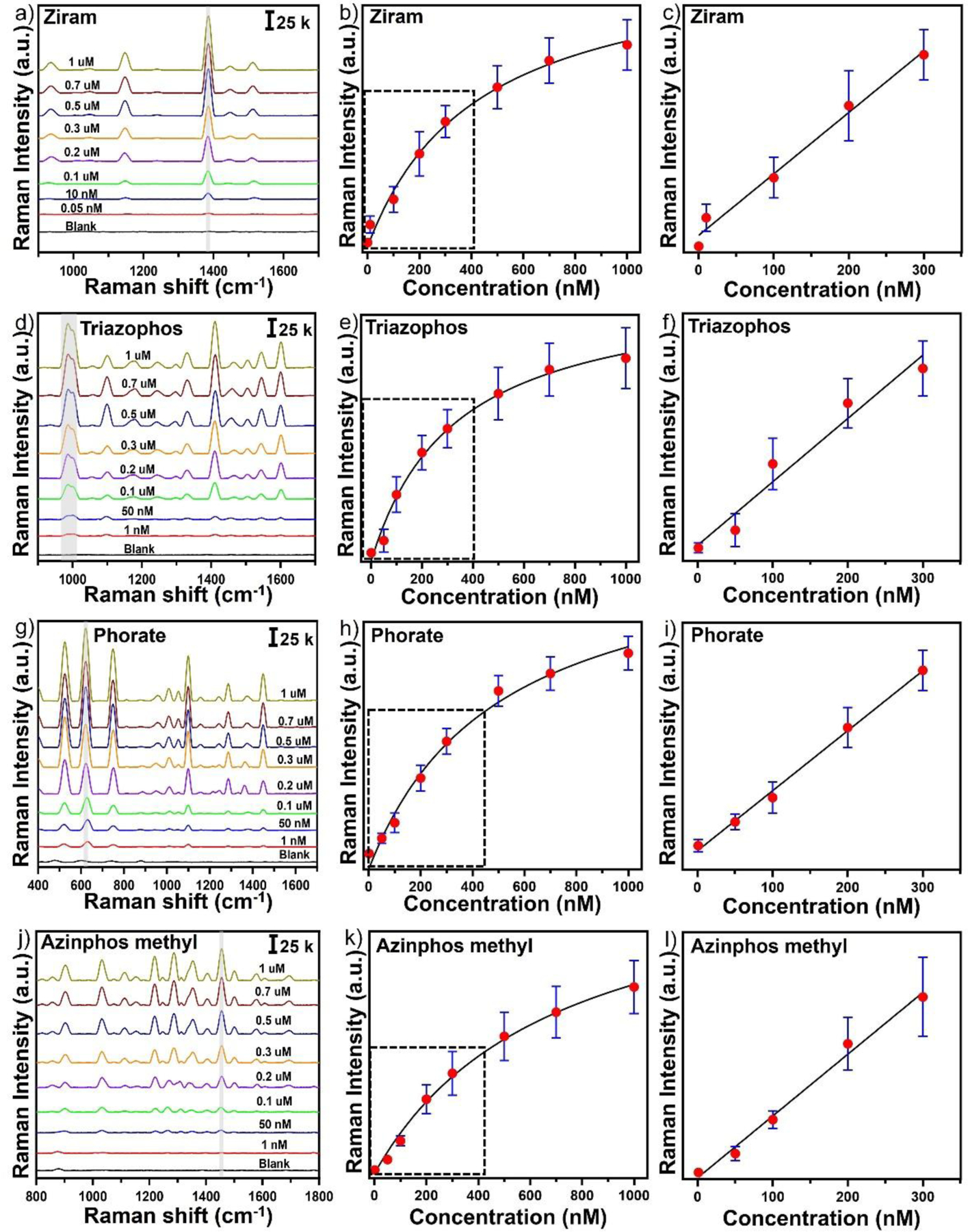
The SERS spectra and the calibration curve of ziram (a-c), triazophos (d-f), phorate (g-i), and azinphos methyl (j-l).

Next, to investigate its feasibility for practical rapid on-site detection, the solution-based SERS substrate with GO-SGNS at an optimal concentration integrated with a portable Raman analyzer was used to detect four pesticides (ziram, phorate, triazophos, and azinphos-methyl) in water. Figure 4a displayed the SERS spectra of the different concentrations of ziram ranging from 1 μM to 50 pM. It is important to note that, even at concentrations as low as picomolar concentration levels, the characteristic strongest SERS peaks of ziram at 1384 cm^−1^ can still be observed with a good signal-to-noise ratio (S/N= 3.5) (Figure 4a). Figure 4b exhibited the calibration curve of ziram, where the SERS intensity at 1384 cm^−1^ versus the concentration of ziram was fitted with a one-site specific binding equation as described below:

$$y=\text{Bmax}\times \frac{x}{Kd+x}$$

Where *y* is the SERS intensity of ziram, *Bmax* is the SERS intensity at the saturation coverage (Bmax= 72806), *Kd* is the equilibrium dissociation constant in the competitive adsorption process (*Kd* =328.1), and *x* is the concentration of ziram. Moreover, Figure 4c displayed a linear relationship between the SERS intensity at 1384 cm^−1^ versus the concentration of ziram in the concentration range from 0.3 μM to 50 pM. According to the calibration curve, the R^2^ value was 0.96. The LOD of ziram was calculated to 10 pM with a good signal-to-noise ratio (S/N= 3.5). The LOD was much lower than most previous reports. We have studied the quantitative detection and calibration curve of the other three pesticides- phorate, triazophos, and azinphos-methyl. The strongest SERS peaks for the phorate, triazophos, and azinphos-methyl occurred at 622, 988, and 1455 cm^−1^, respectively. Interestingly, all of these pesticides exhibited a one-site specific binding equation for the calibration curve at the maximum SERS peak intensity described above for the ziram. They showed a linear relationship in the concentration range. The LOD was achieved as low as 50 pM, 100 pM, and 100 pM for phorate, triazophos, and azinphos-methyl, respectively. Notably, the LOD of the pesticides determined by our solution-based SERS platform using GO-SGNS can satisfy the detection requirements according to the European Union food safety standard^68^. These results demonstrated that the developed solution-based SERS detection platform could be able to simultaneously screen multiple pesticides without sample treatment by detecting their unique and strongest characteristic SERS peaks, underscoring the capability of the SERS system for rapid on-site detection of multiple pesticide screening in actual samples.

In addition, we further validated our SERS method with HPLC analysis. Figure S7 shows the standard HPLC curve of the three other pesticides including phorate, azinphos-methyl, and triazophos. Interestingly, we were unable to detect ziram by using our HPLC detection protocol. We believe that we need further optimization of the HPLC protocol to determine ziram pesticide. In Table S1, we compare the SERS and HPLC methods and find that our simple rapid SERS method is effective in detecting nanomolar concentrations of pesticides in a good percentage of recovery.

### SERS analysis of pesticides on apple surfaces

To further demonstrate direct detection of pesticides in actual samples such as apple surfaces, the apple surface was sprayed with the pesticides. To fabricate the real sample for SERS analysis, the apple surface was cut into 1×1 cm squares and added to the GO-SGNS solution for SERS detection. Notably, the characteristic SERS peak of ziram at 1384 cm^−1^ can still be clearly distinguished with some subtle variation from the interference of the macromolecules such as proteins, organic acids, and sugar in the apple surface. We conducted each measurement three times to reduce significant errors. The SERS measurements take only one second to collect the Raman spectra and the whole testing process took around 4–5 minutes, indicating rapid point-of-care detection. In addition to pesticide residues on the surface, pesticide residues are also present in the flesh of the fruit^69^. Detailed quantitative investigations of pesticides present in fruit flesh will be conducted in future studies. However, this is beyond the scope of this work.

**Figure 5.**
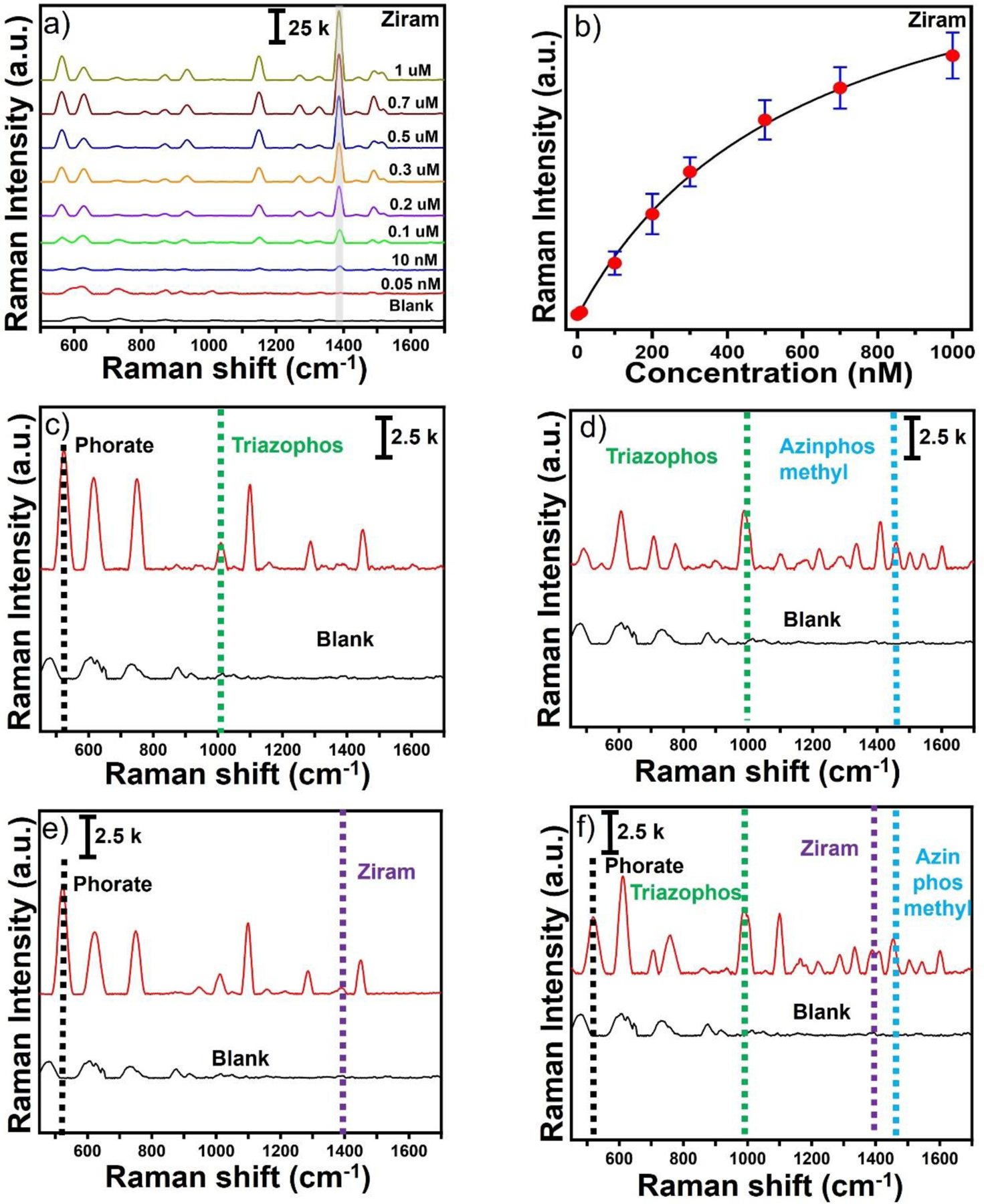
The SERS spectra of the ziram on apple peels where surfaces of apples were pretreated with ziram (a-b). The SERS spectra of binary and quaternary identification of the pesticide molecules: phorate-triazophos (c), triazophos-azinphos-methyl (d), phorate-ziram (e), and phorate-triazophos-azinphos-methyl-ziram mixture where the concentration of the chemicals was 10 nM for ziram and 100 nM for phorate, triazophos, and azinphos-methyl (f).

Figure 5a displayed the SERS spectra of the different concentrations of ziram ranging from 1 μM to 50 pM with a LOD of 10 pM achieved with excellent signal-to-noise ratios. Figure 5b exhibited the calibration curve of ziram, where the SERS intensity at 1384 cm^−1^ versus the concentration of ziram was fitted with a one-site specific binding equation. Compared with previously reported work,^29, 70^ our solution-based SERS platform shows excellent SERS efficiency for detecting ziram without any sample pre-treatment.

An excellent SERS substrate offers the ability to detect multiple pesticides in complex samples, as for real-life agricultural production, multiple pesticides are often used to improve the killing efficiency of pests. Thus, it is important to detect multiple residues on the fruit’s surface. As a proof-of-concept test, we attempted to study the performance of our solution-based SERS substrate using GO-SGNS for the identification capability of binary and quaternary mixtures of pesticides on apple peels. As demonstrated in Figure 5c–e, the SERS spectra of the binary mixed pesticides ziram-phorate, phorate-triazophos, and triazophos-azinphos-methyl were collected from apple peels, where the characteristic peak of the pesticides can provide the information on the concentration and composition of the pesticide mixture. Two pesticides were mixed at equal concentrations at 100 nM for binary detection of pesticides. For quaternary detection of pesticides, all four pesticides were mixed at equal concentrations at 100 nM, indicating a good response to the four pesticide molecules (Figure 5f). It is important to note that the characteristic SERS peaks of triazophos and ziram appear at 1412 cm^−1^ and 1384 cm^−1^, respectively. Therefore, triazophos and ziram generated a doublet peak when detecting the mixture of the four pesticides. Indeed, the peak at 1412 cm^−1^ for triazophos and 1384 cm^−1^ for ziram was clearly distinguishable (Figure 5f). A zoomed in SERS spectra from 1300 to 1600 cm^−1^ of the quaternary pesticides’ mixture (phorate, triazophos, azinphos-methyl, and ziram) indicates that the characteristic SERS peak for ziram at 1384 cm^−1^ clearly differs from the triazophos peak at 1412 cm^−1^ (Figure S8). This showcases the advantage of the SERS technique in showing fingerprint vibrational spectra of multiple analytes with similar chemical compositions. Notably, the SERS intensity of the individual pesticides was slightly decreased for binary and quaternary mixtures than that of individual SERS spectra at 100 nM concentration. We believe that overall SERS enhancement is affected by the affinity of the analytes. Consequently, calibration equations might differ when pesticides are combined in binary or quaternary mixtures. Detailed investigation of calibration equations for quantitative determination of the individual pesticides in the presence of multiple pesticides will be conducted in future studies. However, this is beyond the scope of this work. Moreover, we compared the SERS sensitivity of our GO-SGNS platform with the reported SERS methods for the detection of pesticides. Table S2 summarizes the reported SERS methods of the pesticide’s detection including ziram, azinphos-methyl, phorate, and triazophos which indicates that our SERS substrate shows 100 times superior sensitivity. As a whole, these results demonstrated that our solution-based SERS platform has the potential for rapid and sensitive detection of multiple pesticides in real samples.

In this study, as a demonstration of feasibility, we presented a SERS detection platform utilizing GO-SGNS, tailored for the identification of four specific pesticides: ziram, phorate, triazophos, and azinphos-methyl. However, we acknowledge inherent limitations of our SERS method in its capability to simultaneously detect more than four analytes. While Raman spectroscopy is recognized for offering distinct “spectral fingerprints” of molecules, there exists a chance that analytes with similar chemical compositions and resonance Raman effects may cause certain Raman vibrational bands to overlap. As a result, effectively correlating SERS intensities with analyte concentrations poses challenges. Furthermore, practical limitations emerge when utilizing SERS for the qualitative analysis of pesticides on apple surfaces, especially when multiple pesticides exhibit diverse affinities toward the gold nanoparticle surface. A thorough examination of detecting multiplex pesticides demands meticulous effort and parameter optimization for conducting SERS measurements. It is noteworthy that we have successfully analyzed the SERS data of a multicomponent mixture consisting of five polyaromatic hydrocarbons (PAH) using artificial intelligence (AI) with machine learning algorithms based on the convolutional neural network (CNN) model in order to discriminate the PAH in samples more precisely and accurately^71^. A detailed investigation for quantitative determination of a large number of pesticides will be conducted in future studies, which could include AI-enabled analysis. However, this is beyond the scope of this work.

Our finding indicates that solution-based SERS spectroscopy encounters limitations in quantitative detection of pesticides, mainly due to variations in analyte affinities towards the gold nanoparticle surface. For instance, analytes with greater affinity tend to occupy the ‘hot-spots’ of GO-SGNS preferentially. As a result, other analytes in the sample solution may not undergo SERS enhancement to the same extent as the target analyte with the highest affinity. Specifically, ziram demonstrates significant attraction to the nanostar surface, resulting in a robust SERS signal, while other analytes show lower signal intensity. Thus, the efficacy of solution-based Raman spectroscopy is constrained by the affinity profiles of the target analytes. Future studies will undertake a comprehensive investigation to quantitatively determine a wide range of pesticides.

## CONCLUSION

The current study designed a simple sensitive solution-based SERS active substrate to identify the pesticide residues on apple surfaces. The silver-gold nanostars (SGNS) coated with graphene oxide (GO) were employed as a SERS sensor for the detection of pesticides. The use of GO on SGNS leads to high sensitivity with excellent SERS reproducibility and stability; thus, offering possibilities for point-of-need in-field application. Our solution-based SERS assay shows ultra-high sensitivity of the pesticides in water with a minimum detection limit of up to 10 pM, 50 pM, 100 pM, and 100 pM for ziram, phorate, triazophos, and azinphos-methyl, respectively. In addition, this SERS substrate showed excellent performance for the detection of ziram on apple surface with a LOD of 10 pM. Moreover, the SERS measurements of the four pesticides mixture indicate the multiplex capability of our SERS platform. Overall, our findings open new avenues for highly effective SERS substrates for rapid and reproducible on-site analyte detection, and it offers the ability to realize multiplex detection of pesticides in complex samples. Our SERS platform has been successfully employed for the detection of four pesticides: ziram, phorate, triazophos, and azinphos-methyl. The incorporation of GO with SGNS enhances colloidal stability and ensures reproducibility in SERS detection. While our SERS platform significantly enhances sensitivity for pesticide detection, we acknowledge its limitations. Specifically, this solution-based SERS platform may not be ideal for simultaneously detecting multiple pesticides as they have different affinities towards gold nanoparticle surface. Furthermore, practical constraints may arise when using SERS for the qualitative analysis of pesticides on apple surfaces, particularly when multiple pesticides of the same type, possessing similar resonance Raman effects, are present on the apple surface. Subsequent research endeavors could involve the utilization of artificial intelligence (AI) alongside machine learning algorithms, specifically those leveraging the convolutional neural network (CNN) model, which could facilitate the accurate distinction of Raman peaks and the identification of diverse pesticides. Furthermore, solution-based SERS faces challenges in achieving quantitative multiplex detection of pesticides, potentially stemming from variations in analyte affinities toward the gold nanoparticle surface. Subsequent studies will conduct a thorough investigation to quantitatively determine multiple pesticides.

## Supplementary Material

Supporting Information

The Supporting Information is available free of charge.

Details of HPLC methodology, TEM images of GNS, SGNS, DLS size distribution of GO-SGNS, TEM images of GO-SGNS’, and GO-SGNS”, blank SERS spectra of GO-SGNS at different concentrations of GO loaded with SGNS, SERS intensity comparison of GO-SGNSs with different thickness of silver on GNS, the Raman and SERS spectrum of TP at 100 mM concentration, SERS intensity of the peak at 1075 cm^−1^ of TP for 30 different spots, HPLC standard curve of phorate, azinphos methyl, and triazophos, zoomed in SERS spectra from 1300 to 1600 cm^−1^ of the quaternary pesticides’ mixture (phorate, triazophos, azinphos-methyl, and ziram), comparison for detecting phorate, azinphos methyl, and triazophos using SERS technique and HPLC method, and literature survey for detecting ziram, phorate, azinphos methyl, and triazophos using different SERS techniques.

## Figures and Tables

**Table 1.** Literature survey for AEF of different colloidal SERS substrate.

Colloidal SERS substrate	Analytical enhancement factor (AEF)	Ref.
Gold nanostars	2 ×10^5^	60
Gold nanospheres	5×10^4^	60
Spiny gold nanoparticles	1.8 × 10^4^	61
Porous gold nanoparticles	(1.23 ± 0.10) × 10^7^	62
Fe_3_O_4/_Au Nanoparticles	6.74 × 10^6^	63
Polystyrene sulfonate modified gold nanobipyramids	1.8 × 10^7^	64
HEPES and EPPS buffers mediated gold nanostars	5.2 × 10^5^	65
**GO-SGNS**	**3.2 ×10** ^**8**^	**This work**
