# Supplementary material for: Rapid Solution-Based SERS Detection of Pesticides Using Graphene Oxide-Coated Silver–Gold Nanostars
Source: ACS Appl Nano Mater. Author manuscript; Available in PMC 2026 Jan 13. (PMC12794728; doi:10.1021/acsanm.4c01122)
Supplement: Supporting Information [file NIHMS2126899-supplement-Supporting_Information.docx]

**Supporting formation**

**Rapid Solution-based SERS** **Detection of Pesticides Using Graphene Oxide Coated Silver-Gold Nanostars**

*Supriya Atta, ^a, b^ Tamer Sharaf, ^a, b, c^ Tuan Vo-Dinh ^a,^ ^b, d‡^*

^a^ Fitzpatrick Institute for Photonics, ^b^ Department of Biomedical Engineering, ^c^ Department of Physics, Ain Shams University, Cairo, Egypt, ^d^ Department of Chemistry, Duke University, Durham, NC 27708, USA.

‡ Correspondence author: tuan.vodinh@duke.edu

**Experimental section**

**High-performance Liquid Chromatography Mass Spectrometry (LCMS):** LCMS analysis was performed on a 6224 TOF LC/MS system (Agilent Technologies), consisting of a 1200 HPLC (degasser, binary pump, thermostated column compartment, diode array detector (DAD)) coupled to a 6224 accurate-mass time-of-flight mass spectrometer. The mass spectrometer was equipped with a Dual ESI source, and accurate mass data were obtained by internal calibration (reference ion 922.009798 m/z) using a secondary nebulizer to continuously deliver the reference solution. Positive-ion mass spectral data were acquired in full-scan mode over the range of 60-1700 m/z using the following source parameters: gas temperature 325 °C, gas flow 11 L/min, nebulizer pressure 33 psig, VCap 3500 V, and fragmentor voltage 150 V. HPLC separations were achieved on a Phenomenex Kinetix C18 column (3 x 30 mm, 2.6 µ) using a linear gradient of mobile phase B in A, a flow rate of 0.5 mL/min, and a column temperature of 40 °C. Mobile phase A was prepared by combining 400 mL ultrapure water with 12 mL methanol and 1.2 mL formic acid. Mobile phase B was prepared by combining 400 mL acetonitrile with 12 mL ultrapure water and 1.2 mL formic acid. The gradient program included an initial hold at 10% solvent B for 0.5 min, followed by a linear increase to 50% solvent B from 0.5-7 min, then a linear increase from 50-100% B from 7-8.1 min, a hold at 100% solvent B from 8.1-9 min, and re-equilibration back to 0% B for a total run time of 15 min. Samples were analyzed using a 5-µL injection volume. The elution order of the pesticides was azinphos-methyl (8.4 min), followed by triazophos (9.4 min), followed by phorate (10.0 min). Peak areas were integrated from extracted ion chromatograms of the [M+H]+ ions of each compound (318.01 m/z for azinphos-methyl, 314.07 m/z for triazophos, and 261.02 m/z for phorate). It is important to note that we were unable to detect ziram with our HPLC method as ziram is partially soluble in methanol.


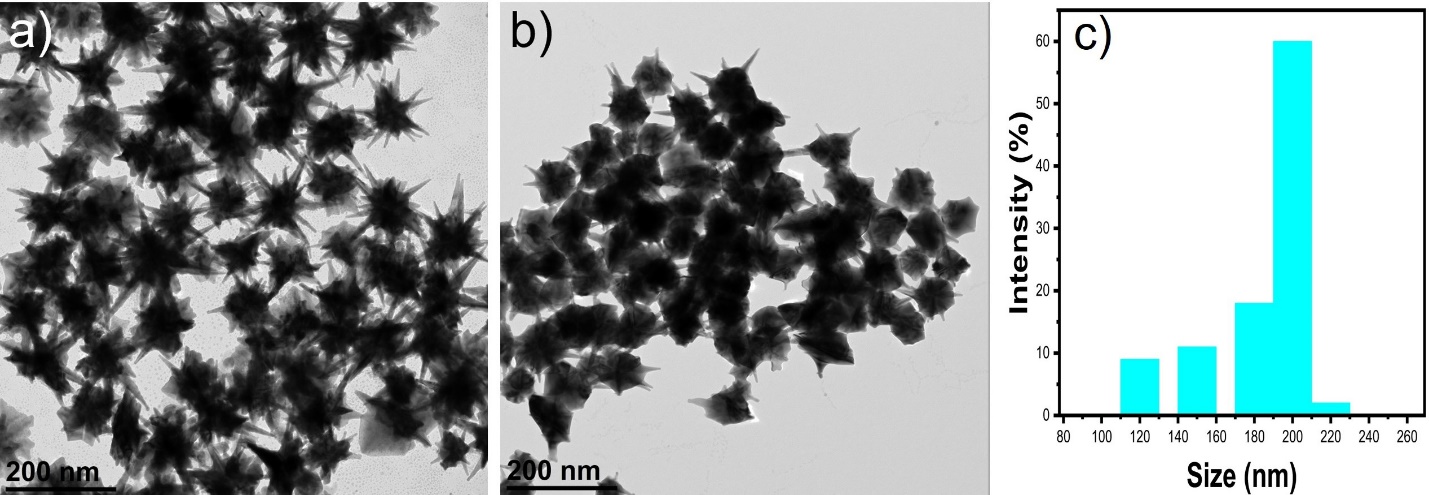


**Figure S1.** TEM images of GNS (a) and SGNS (b) exhibiting highly monodispersed morphology of the nanostars. DLS size distribution of GO-SGNS (c).


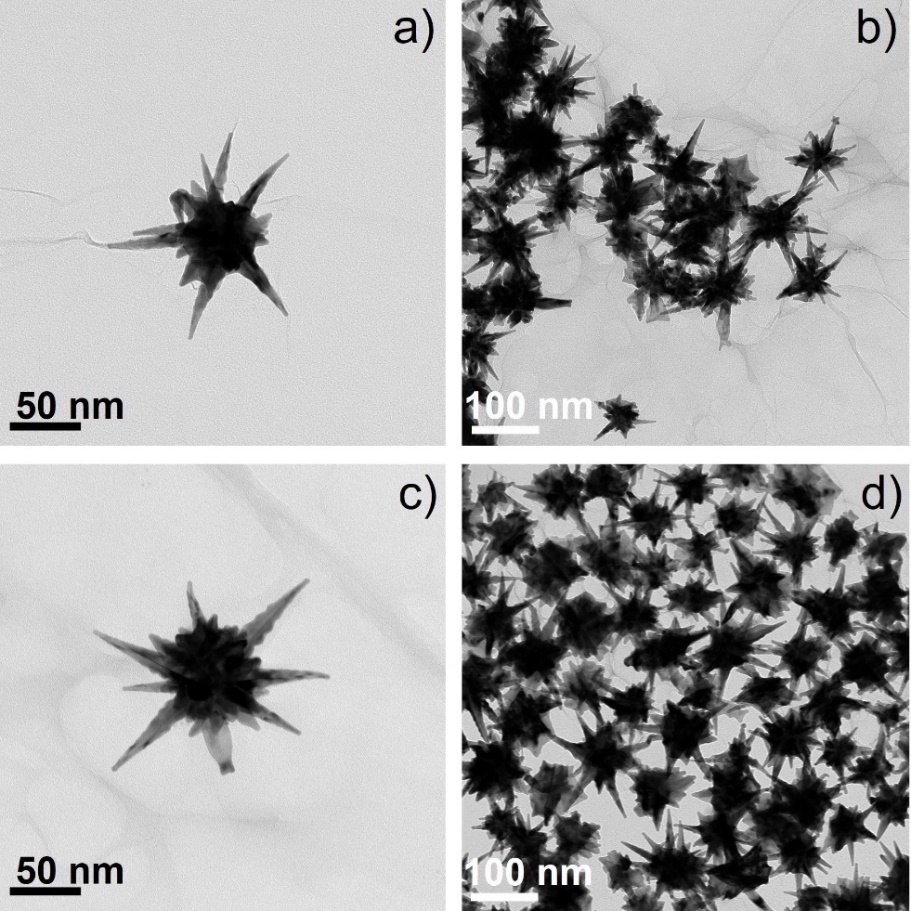


**Figure S2.** TEM images of GO-SGNS' (a-b) and GO-SGNS" exhibit highly monodispersed morphology of the nanostars (c-d).


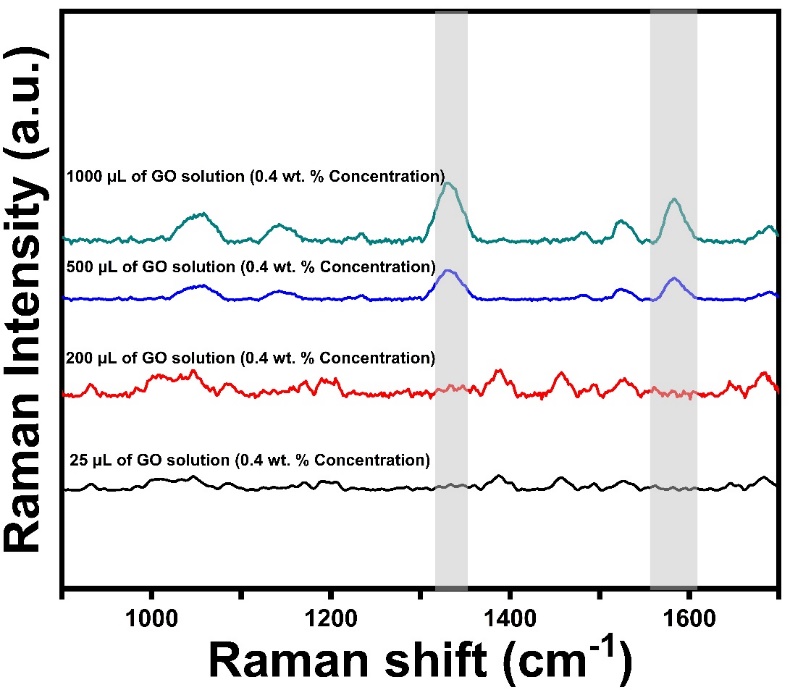


**Figure S3.** The blank SERS spectra of GO-SGNS at different concentrations of GO loaded with SGNS indicating that the characteristic SERS signal of GO at 1330 cm^-1^ and 1584 cm^-1^ increased with increased GO loading.


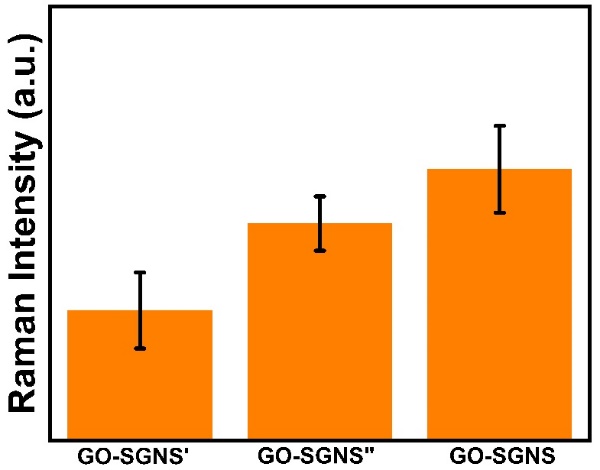


**Figure S4.** SERS intensity comparison of GO-SGNSs with different thickness of silver on GNS.


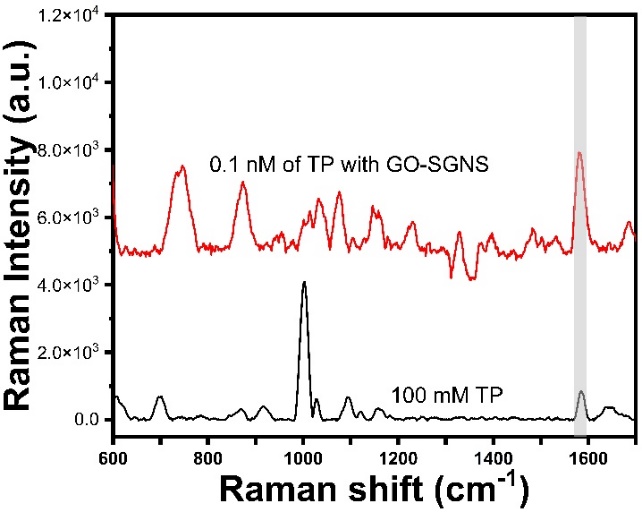


**Figure S5.** The Raman spectrum of TP at 100 mM concentration (black) and the SERS spectra of TP at 0.1 nM concentration (red).


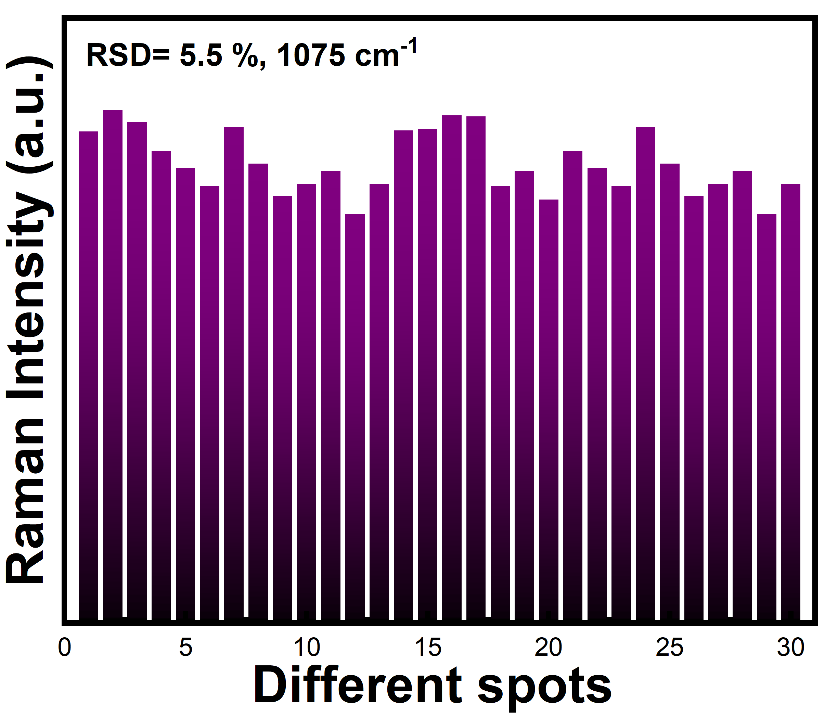


**Figure S6.** SERS intensity of the peak at 1075 cm^-1^ of TP for 30 different spots.


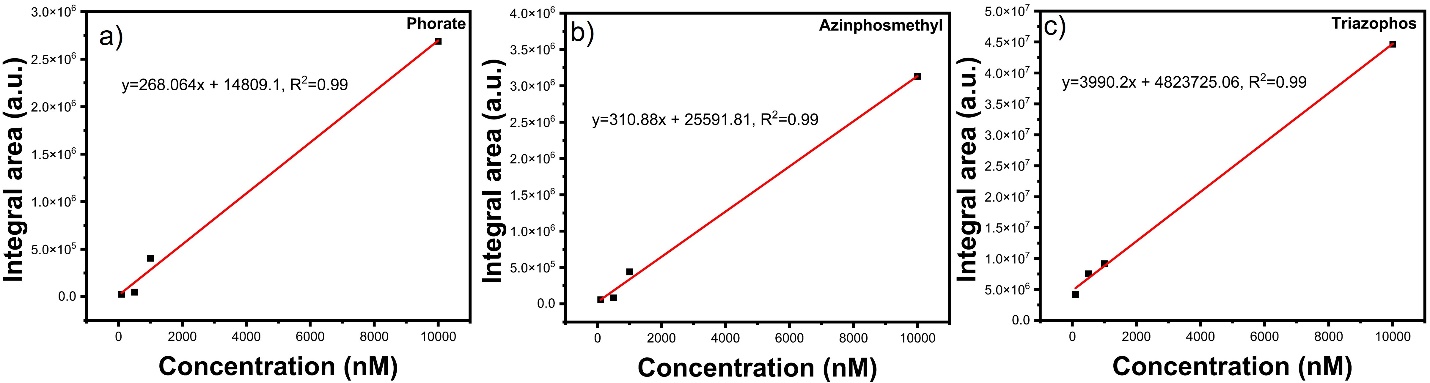
**Figure S7.** High-performance liquid chromatography (HPLC) standard curve of phorate, azinphos methyl, and triazophos (a-c).


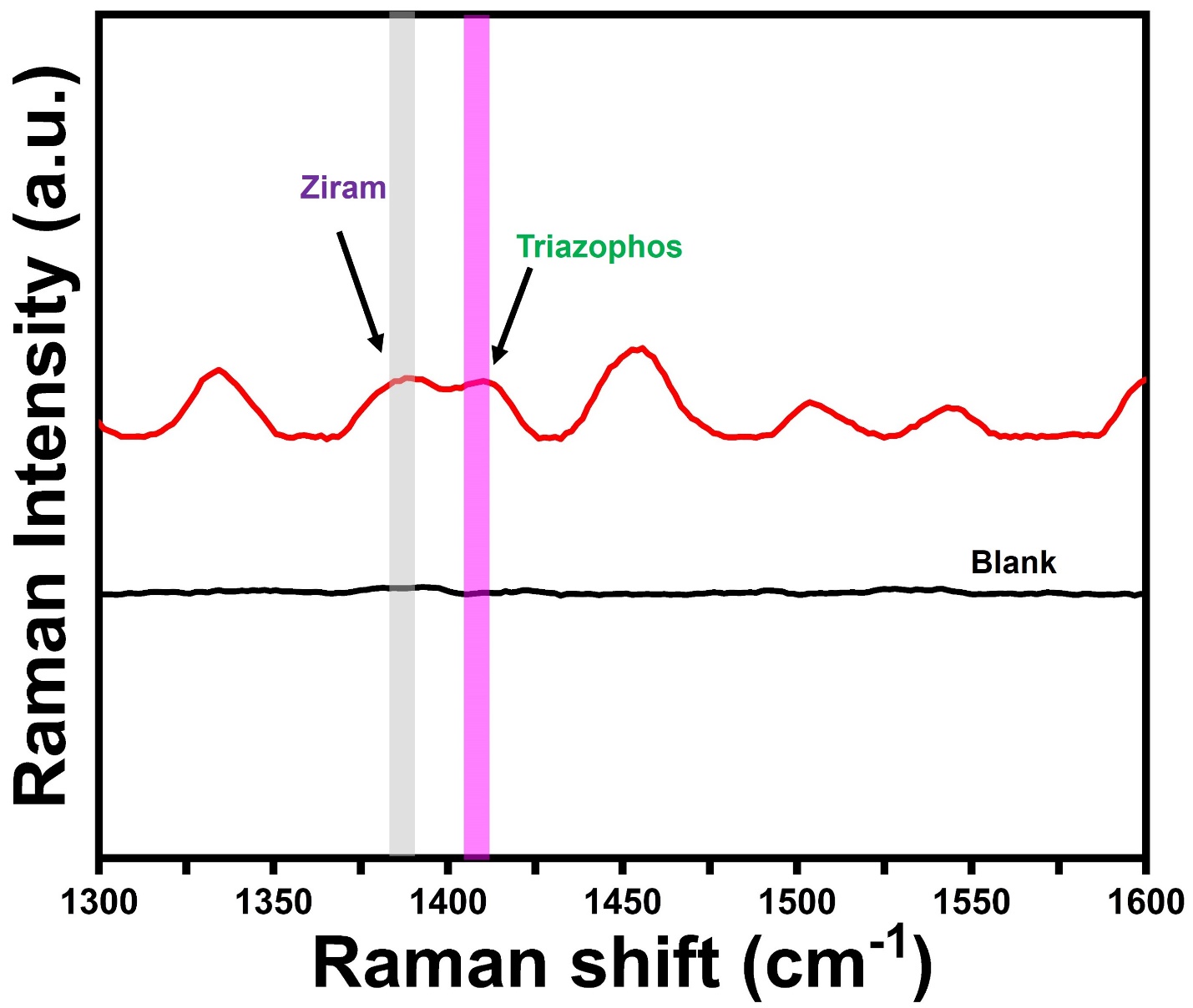


**Figure S8.** A zoomed in SERS spectra from 1300 to 1600 cm^-1^ of the quaternary pesticides’ mixture (phorate, triazophos, azinphos-methyl, and ziram) indicating that the characteristic SERS peak for ziram at 1384 cm^-1^ and for triazophos at 1412 cm^-1^ can be clearly distinguishable.

**Table S1.** Comparison for detecting phorate, azinphos methyl, and triazophos using SERS technique and HPLC method.

| Pesticides | \| Spiked (nM) \|  \| \| --- \| --- \| | |  | Detected concentration by SER | Recovery (%) |  | Detected concentration by HPLC (nM) | |  | | Recovery (%) | |
| --- | --- | --- | --- | --- | --- | --- | --- | --- | --- | --- | --- | --- | --- | --- |
| Phorate | 1000 | |  | 998.20 | 99.82 |  | 1155.3 | |  | | 115.5 | |
|  | 100 | |  | 90.56 | 90.56 |  | 86.6 | |  | | 86.6 | |
|  | 1 | |  | 1.03 | 103 |  | - | |  | | - | |
| Azinphos methyl | 1000 | |  | 869.52 | 86.95 |  | 1165 | |  | | 116.5 | |
|  | 100 | |  | 93.20 | 93.2 |  | 103.1 | |  | | 103.1 | |
|  | 1 | |  | 0.93 | 93 |  | - | |  | | - | |
| Triazophos | 1000 | |  | 1010.3 | 101 |  | 1106 | |  | | 110.6 | |
|  | 100 | |  | 110.4 | 110.4 |  | 85.6 | |  | | 85.6 | |
|  | 1 | |  | 0.97 | 97 |  | - | |  | | - | |
|  | |  | | | | | |  | |  | |  |

**Table S2.** Literature survey for detecting ziram, phorate, azinphos methyl, and triazophos using different SERS techniques.

| Pesticides | SERS Substrate | LOD | Reference |
| --- | --- | --- | --- |
| Ziram | Gold nanorods | 12.94 nM | ^1^ |
| Ziram | Octanethiol functionalized Au@AgNPs | ~49 µM | ^2^ |
| Ziram | PAMAM mediated Ag:Au nanostructures | 100 nM | ^3^ |
| Triazophos | MOF-induced ZnO@Co_3_O_4_ modified AgNPs | 1 nM | ^4^ |
| Phorate | Stainless steel wires coated with gold (Au) nanoparticles | 97 µM | ^5^ |
| Phorate | Aptamer based silver (Ag) dendrites | 24 μM | ^6^ |
| Phorate | Silver nanoplate-decorated copper membranes | 3.84 nmol L^-1^ | ^7^ |
| Azinphos methyl | Graphene-gold film-gold nanorod | 15.77 µM | ^8^ |
| Azinphos methyl | Gold-coated SERS-active nano substrates | 21 µM | ^9^ |
| Ziram | **GO-SGNS (this work)** | **10 pM** | **-** |
| Triazophos | **GO-SGNS (this work)** | **100 pM** | **-** |
| Phorate | **GO-SGNS (this work)** | **50 pM** | **-** |
| Azinphos methyl | **GO-SGNS (this work)** | **100 pM** | **-** |

**References**

1. Saute, B.; Premasiri, R.; Ziegler, L.; Narayanan, R., Gold nanorods as surface enhanced Raman spectroscopy substrates for sensitive and selective detection of ultra-low levels of dithiocarbamate pesticides. *Analyst* **2012,** *137* (21), 5082-5087.

2. Hussain, N.; Pu, H.; Hussain, A.; Sun, D.-W., Rapid detection of ziram residues in apple and pear fruits by SERS based on octanethiol functionalized bimetallic core-shell nanoparticles. *Spectrochimica Acta Part A: Molecular and Biomolecular Spectroscopy* **2020,** *236*, 118357.

3. Fernandes, T.; Fateixa, S.; Ferro, M.; Nogueira, H. I. S.; Daniel-da-Silva, A. L.; Trindade, T., Colloidal dendritic nanostructures of gold and silver for SERS analysis of water pollutants. *Journal of Molecular Liquids* **2021,** *337*, 116608.

4. Ye, C.; He, M.; Zhu, Z.; Shi, X.; Zhang, M.; Bao, Z.; Huang, Y.; Jiang, C.; Li, J.; Wu, Y., A portable SERS sensing platform for the multiplex identification and quantification of pesticide residues on plant leaves. *Journal of Materials Chemistry C* **2022,** *10* (36), 12966-12974.

5. Wang, C.; Zhang, Z.; He, L., Development of a headspace solid-phase microextraction–surface-enhanced Raman scattering approach to detect volatile pesticides. *Journal of Raman Spectroscopy* **2019,** *50* (1), 6-14.

6. Pang, S.; Labuza, T. P.; He, L., Development of a single aptamer-based surface enhanced Raman scattering method for rapid detection of multiple pesticides. *Analyst* **2014,** *139* (8), 1895-1901.

7. Yu, X.; Chang, Y.; Natarajan, V.; Zhang, X.; Zhan, J., Recyclable silver nanoplate-decorated copper membranes for solid-phase extraction coupled with surface-enhanced Raman scattering detection. *Analytical Methods* **2018,** *10* (11), 1353-1361.

8. Nguyen, T. H. D.; Zhang, Z.; Mustapha, A.; Li, H.; Lin, M., Use of Graphene and Gold Nanorods as Substrates for the Detection of Pesticides by Surface Enhanced Raman Spectroscopy. *Journal of Agricultural and Food Chemistry* **2014,** *62* (43), 10445-10451.

9. Liu, B.; Zhou, P.; Liu, X.; Sun, X.; Li, H.; Lin, M., Detection of Pesticides in Fruits by Surface-Enhanced Raman Spectroscopy Coupled with Gold Nanostructures. *Food and Bioprocess Technology* **2013,** *6* (3), 710-718.
